# Supplementary figures and images for: Survival outcomes of breast cancer patients with recurrence after surgery according to period and subtype
Source: PLoS One. 2023 Jul 27;18(7):e0284460. doi: 10.1371/journal.pone.0284460 (PMC10374104; doi:10.1371/journal.pone.0284460)

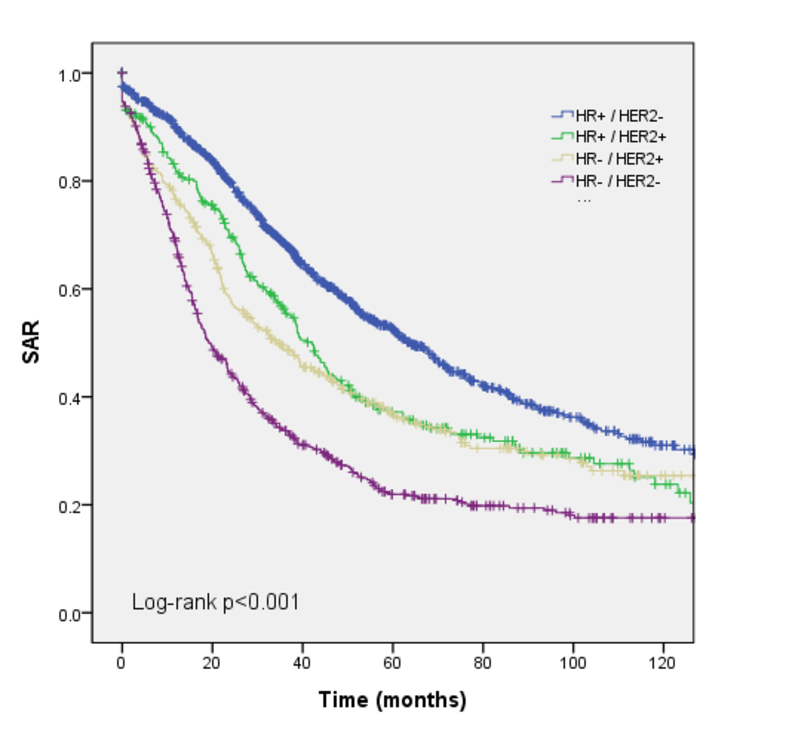

Supplement: S1 Fig — (TIF) [file pone.0284460.s001.tif]

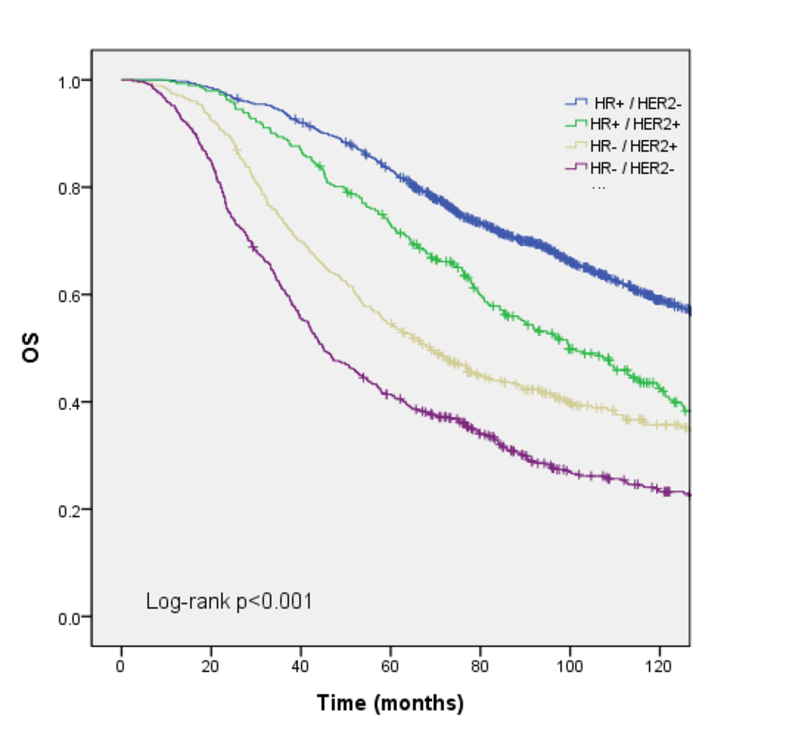

Supplement: S2 Fig — (TIF) [file pone.0284460.s002.tif]
