# Supplementary material for: Survival outcomes of breast cancer patients with recurrence after surgery according to period and subtype
Source: PLoS One. 2023 Jul 27;18(7):e0284460. doi: 10.1371/journal.pone.0284460 (PMC10374104; doi:10.1371/journal.pone.0284460)
Supplement: S1 Table — (DOCX) [file pone.0284460.s003.docx]

| Factors | Survival after recurrence | | | Overall survival | | |
| --- | --- | --- | --- | --- | --- | --- |
|  | HR | 95% CI | p-value | HR | 95% CI | p-value |
| Year of diagnosis |  |  |  |  |  |  |
| 2000–2007 | 1.00 | Ref. |  | 1.00 | Ref. |  |
| 2008–2013 | 0.71 | 0.58–0.86 | <0.001 | 0.85 | 0.70–1.04 | 0.122 |
| Age at diagnosis (y) |  |  | <0.001 |  |  | <0.001 |
| 35–50 | 1.00 | Ref. |  | 1.00 | Ref. |  |
| <35 | 0.97 | 0.74–1.28 | 0.843 | 0.83 | 0.63–1.09 | 0.177 |
| >50 | 1.75 | 1.32–2.33 | <0.001 | 1.38 | 1.10–1.95 | 0.008 |
| T stage |  |  | <0.001 |  |  | <0.001 |
| T1 | 1.00 | Ref. |  | 1.00 | Ref. |  |
| T2 | 1.70 | 1.38–2.08 | <0.001 | 1.83 | 1.49–2.25 | <0.001 |
| T3 | 3.13 | 2.35–4.18 | <0.001 | 3.71 | 2.78–4.96 | <0.001 |
| T4 | 4.71 | 2.97–7.45 | <0.001 | 6.26 | 3.95–9.94 | <0.001 |
| Nodal stage |  |  |  |  |  |  |
| Negative | 1.00 | Ref. |  | 1.00 | Ref. |  |
| Positive | 2.34 | 1.92–2.84 | <0.001 | 2.55 | 2.10–3.09 | <0.001 |
| Histologic grade |  |  | <0.001 |  |  | <0.001 |
| G1 | 1.00 | Ref. |  | 1.00 | Ref. |  |
| G2 | 3.43 | 1.42–8.31 | 0.006 | 4.65 | 1.92–11.27 | 0.001 |
| G3 | 4.91 | 2.02–11.95 | <0.001 | 7.26 | 2.98–17.68 | <0.001 |
| LVI |  |  |  |  |  |  |
| No | 1.00 | Ref. |  | 1.00 | Ref. |  |
| Yes | 1.40 | 1.15–1.71 | 0.001 | 1.58 | 1.30–1.92 | <0.001 |
| Breast surgery |  |  |  |  |  |  |
| BCS | 1.00 | Ref. |  | 1.00 | Ref. |  |
| TM | 1.96 | 1.61–2.39 | <0.001 | 2.00 | 1.64–2.43 | <0.001 |
| Chemotherapy after recurrence |  |  |  |  |  |  |
| No | 1.00 | Ref. |  | 1.00 | Ref. |  |
| Yes | 2.88 | 2.36–3.52 | <0.001 | 3.22 | 2.64–3.94 | <0.001 |
| Anti-hormonal therapy after recurrence |  |  |  |  |  |  |
| No | 1.00 | Ref. |  | 1.00 | Ref. |  |
| Yes | 0.83 | 0.64–1.06 | 0.143 | 0.93 | 0.72–1.19 | 0.547 |
| Anti-targeted therapy after recurrence |  |  |  |  |  |  |
| No | 1.00 | Ref. |  | 1.00 | Ref. |  |
| Yes | 0.96 | 0.70–1.31 | 0.777 | 0.85 | 0.62–1.16 | 0.295 |
