# Supplementary material for: Survival outcomes of breast cancer patients with recurrence after surgery according to period and subtype
Source: PLoS One. 2023 Jul 27;18(7):e0284460. doi: 10.1371/journal.pone.0284460 (PMC10374104; doi:10.1371/journal.pone.0284460)
Supplement: S2 Table — (DOCX) [file pone.0284460.s004.docx]

| Factors | Survival after recurrence | | | Overall survival | | |
| --- | --- | --- | --- | --- | --- | --- |
|  | HR | 95% CI | p-value | HR | 95% CI | p-value |
| Year of diagnosis |  |  |  |  |  |  |
| 2000–2007 | 1.00 | Ref. |  | 1.00 | Ref. |  |
| 2008–2013 | 0.56 | 0.40–0.78 | 0.001 | 0.79 | 0.56–1.11 | 0.178 |
| Age at diagnosis (y) |  |  | 0.116 |  |  | 0.201 |
| 35–50 | 1.00 | Ref. |  | 1.00 | Ref. |  |
| <35 | 0.81 | 0.53–1.24 | 0.334 | 0.90 | 0.59–1.37 | 0.614 |
| >50 | 1.14 | 0.72–1.81 | 0.567 | 1.22 | 0.77–1.93 | 0.405 |
| T stage |  |  | 0.001 |  |  | <0.001 |
| T1 | 1.00 | Ref. |  | 1.00 | Ref. |  |
| T2 | 1.26 | 0.89–1.78 | 0.185 | 1.33 | 0.94–1.88 | 0.108 |
| T3 | 1.20 | 0.72–2.00 | 0.485 | 1.51 | 0.91–2.52 | 0.112 |
| T4 | 3.11 | 1.84–5.25 | <0.001 | 6.16 | 3.61–10.50 | <0.001 |
| Nodal stage |  |  |  |  |  |  |
| Negative | 1.00 | Ref. |  | 1.00 | Ref. |  |
| Positive | 1.96 | 1.42–2.69 | <0.001 | 2.30 | 1.67–3.16 | <0.001 |
| Histologic grade |  |  | 0.826 |  |  | 0.290 |
| G1 | 1.00 | Ref. |  | 1.00 | Ref. |  |
| G2 | 0.81 | 0.33–2.01 | 0.654 | 0.92 | 0.37–2.27 | 0.850 |
| G3 | 0.88 | 0.36–2.17 | 0.782 | 1.18 | 0.48–2.91 | 0.727 |
| LVI |  |  |  |  |  |  |
| No | 1.00 | Ref. |  | 1.00 | Ref. |  |
| Yes | 1.223 | 0.87–1.73 | 0.254 | 1.49 | 1.05–2.11 | 0.024 |
| Breast surgery |  |  |  |  |  |  |
| BCS | 1.00 | Ref. |  | 1.00 | Ref. |  |
| TM | 1.79 | 1.27–2.53 | 0.001 | 1.69 | 1.19–2.38 | 0.003 |
| Chemotherapy after recurrence |  |  |  |  |  |  |
| No | 1.00 | Ref. |  | 1.00 | Ref. |  |
| Yes | 2.42 | 1.69–3.46 | <0.001 | 2.67 | 1.86–3.82 | <0.001 |
| Anti-hormonal therapy after recurrence |  |  |  |  |  |  |
| No | 1.00 | Ref. |  | 1.00 | Ref. |  |
| Yes | 0.78 | 0.57–1.08 | 0.137 | 0.92 | 0.67–1.27 | 0.599 |
| Anti-targeted therapy after recurrence |  |  |  |  |  |  |
| No | 1.00 | Ref. |  | 1.00 | Ref. |  |
| Yes | 1.26 | 0.93–1.73 | 0.139 | 1.46 | 1.07–2.00 | 0.017 |
