# Supplementary material for: Survival outcomes of breast cancer patients with recurrence after surgery according to period and subtype
Source: PLoS One. 2023 Jul 27;18(7):e0284460. doi: 10.1371/journal.pone.0284460 (PMC10374104; doi:10.1371/journal.pone.0284460)
Supplement: S3 Table — (DOCX) [file pone.0284460.s005.docx]

| Factors | Survival after recurrence | | | Overall survival | | |
| --- | --- | --- | --- | --- | --- | --- |
|  | HR | 95% CI | p-value | HR | 95% CI | p-value |
| Year of diagnosis |  |  |  |  |  |  |
| 2000–2007 | 1.00 | Ref |  | 1.00 | Ref |  |
| 2008–2013 | 0.59 | 0.45–0.76 | <0.001 | 0.60 | 0.46–0.78 | <0.001 |
| Age at diagnosis (y) |  |  | 0.280 |  |  | 0.285 |
| 35–50 | 1.00 | Ref |  | 1.00 | Ref |  |
| <35 | 0.99 | 0.64–1.51 | 0.948 | 0.99 | 0.65–1.52 | 0.961 |
| >50 | 1.22 | 0.79–1.87 | 0.369 | 1.22 | 0.79–1.88 | 0.365 |
| T stage |  |  | <0.001 |  |  | <0.001 |
| T1 | 1.00 | Ref |  | 1.00 | Ref |  |
| T2 | 2.95 | 2.13–4.09 | <0.001 | 3.15 | 2.27–4.37 | <0.001 |
| T3 | 3.71 | 2.28–6.04 | <0.001 | 4.01 | 2.46–6.51 | <0.001 |
| T4 | 5.22 | 3.21–8.50 | <0.001 | 6.31 | 3.88–10.26 | <0.001 |
| Nodal stage |  |  |  |  |  |  |
| Negative | 1.00 | Ref |  | 1.00 | Ref |  |
| Positive | 3.12 | 2.34–4.15 | <0.001 | 3.24 | 2.42–4.28 | <0.001 |
| Histologic grade |  |  | 0.023 |  |  | 0.010 |
| G1 | 1.00 | Ref |  | 1.00 | Ref |  |
| G2 |  |  | 0.811 |  |  | 0.807 |
| G3 |  |  | 0.806 |  |  | 0.800 |
| LVI |  |  |  |  |  |  |
| No | 1.00 | Ref |  | 1.00 | Ref |  |
| Yes | 1.93 | 1.46–2.55 | <0.001 | 2.05 | 1.56–2.71 | <0.001 |
| Breast surgery |  |  |  |  |  |  |
| BCS | 1.00 | Ref |  | 1.00 | Ref |  |
| TM | 2.10 | 1.55–2.83 | <0.001 | 2.30 | 1.70–3.10 | <0.001 |
| Chemotherapy after recurrence |  |  |  |  |  |  |
| No | 1.00 | Ref. |  | 1.00 | Ref. |  |
| Yes | 2.48 | 1.83–3.37 | <0.001 | 2.40 | 1.76–3.25 | <0.001 |
| Anti-hormonal therapy after recurrence |  |  |  |  |  |  |
| No | 1.00 | Ref. |  | 1.00 | Ref. |  |
| Yes | 0.88 | 0.60–1.30 | 0.520 | 0.85 | 0.58–1.26 | 0.424 |
| Anti-targeted therapy after recurrence |  |  |  |  |  |  |
| No | 1.00 | Ref. |  | 1.00 | Ref. |  |
| Yes | 1.27 | 0.97–1.66 | 0.084 | 1.30 | 1.00–1.71 | 0.055 |
