# Supplementary material for: Survival outcomes of breast cancer patients with recurrence after surgery according to period and subtype
Source: PLoS One. 2023 Jul 27;18(7):e0284460. doi: 10.1371/journal.pone.0284460 (PMC10374104; doi:10.1371/journal.pone.0284460)
Supplement: S4 Table — (DOCX) [file pone.0284460.s006.docx]

| Factors | Survival after recurrence | | | Overall survival | | |
| --- | --- | --- | --- | --- | --- | --- |
|  | HR | 95% CI | p-value | HR | 95% CI | p-value |
| Year of diagnosis |  |  |  |  |  |  |
| 2000–2007 | 1.00 | Ref. |  | 1.00 | Ref. |  |
| 2008–2013 | 0.87 | 0.72–1.05 | 0.140 | 1.00 | 0.83–1.21 | 0.977 |
| Age at diagnosis (y) |  |  | 0.305 |  |  | 0.294 |
| 35–50 | 1.00 | Ref. |  | 1.00 | Ref. |  |
| <35 | 1.01 | 0.75–1.38 | 0.934 | 0.91 | 0.67–1.23 | 0.530 |
| >50 | 1.18 | 0.86–1.63 | 0.306 | 1.07 | 0.78–1.47 | 0.693 |
| T stage |  |  | <0.001 |  |  | <0.001 |
| T1 | 1.00 | Ref. |  | 1.00 | Ref. |  |
| T2 | 1.41 | 1.12–1.79 | 0.004 | 1.57 | 1.24–1.99 | <0.001 |
| T3 | 2.46 | 1.79–3.38 | <0.001 | 2.82 | 2.06–3.87 | <0.001 |
| T4 | 2.59 | 1.73–3.88 | <0.001 | 3.65 | 2.44–5.47 | <0.001 |
| Nodal stage |  |  |  |  |  |  |
| Negative | 1.00 | Ref. |  | 1.00 | Ref. |  |
| Positive | 1.82 | 1.49–2.21 | <0.001 | 1.88 | 1.55–2.29 | <0.001 |
| Histologic grade |  |  |  |  |  |  |
| G1 | - | - |  | - | - |  |
| G2 | 1.00 | Ref. |  | 1.00 | Ref |  |
| G3 | 1.39 | 1.11–1.73 | 0.003 | 1.51 | 1.21–1.89 | <0.001 |
| LVI |  |  |  |  |  |  |
| No | 1.00 | Ref. |  | 1.00 | Ref. |  |
| Yes | 1.74 | 1.43–2.13 | <0.001 | 1.88 | 1.54–2.29 | <0.001 |
| Breast surgery |  |  |  |  |  |  |
| BCS | 1.00 | Ref |  | 1.00 | Ref. |  |
| TM | 1.60 | 1.32–1.94 | <0.001 | 1.71 | 1.41–2.07 | <0.001 |
| Chemotherapy after recurrence |  |  |  |  |  |  |
| No | 1.00 | Ref. |  | 1.00 | Ref. |  |
| Yes | 2.19 | 1.72–2.79 | <0.001 | 2.50 | 1.96–3.17 | <0.001 |
| Anti-hormonal therapy after recurrence |  |  |  |  |  |  |
| No | 1.00 | Ref. |  | 1.00 | Ref. |  |
| Yes | 0.73 | 0.53–1.03 | 0.070 | 0.69 | 0.50–0.97 | 0.031 |
| Anti-targeted therapy after recurrence |  |  |  |  |  |  |
| No | 1.00 | Ref. |  | 1.00 | Ref. |  |
| Yes | 0.81 | 0.52–1.28 | 0.367 | 0.73 | 0.46–1.14 | 0.167 |
