# Supplementary material for: Survival outcomes of breast cancer patients with recurrence after surgery according to period and subtype
Source: PLoS One. 2023 Jul 27;18(7):e0284460. doi: 10.1371/journal.pone.0284460 (PMC10374104; doi:10.1371/journal.pone.0284460)
Supplement: Attachment — Submitted filename: comments_06092022.docx [file pone.0284460.s008.docx]

# Review for PLOS ONE

**Sept 5, 2022**

**Title: Survival outcomes of breast cancer patients with recurrence after surgery according to period and subtype**

This paper presents a study to analyze and compare the survival rates of recurrent breast cancer patients in Korea between two periods (2000–2007; 2008–2013) and to identify the factors associated with outcomes and changes over time in the duration of survival after recurrence. However, there are questions that limit my enthusiasm of the paper, as outlined below.

1. Authors include the patients with unknown clinical variables e.g., stage, histology, etc. Why not removing these patients? If for any clinical and biological reason, this group named as “unknown” is important and would like to assess the effect of that in analyses, still not correct analysis was applied. Authors considered chi-squared method to do the association which will have low power in this scenario, and the non-parametric Fisher exact test is the correct method to be applied.
2. Authors did mention that multivariate Cox model was applied, and I couldn’t follow in which step this method was applied. I already saw the findings using univariate Cox model.
3. Please keep the HR estimate along with p value to report findings.
4. Table 3: Please add appropriate test to assess the association between type of recurrence and year of recurrence.
5. Figures 2 and 3: How about adding supplementary figures to show the KM and log-rank test using OS across all four subtypes and the same test for SAR.
6. Tables (e.g., 4 and 5), for example for some categorical variables e.g., age, T stage, etc., we have the p values and other statistics for each category, while a p value for that given variable (e.g., age, T stage). I couldn’t follow how the p value was computed.
7. In addition, authors only considered clinical data, while adding molecular data along with clinical data to assess the association with OS or SAR can make the paper much more interesting along with this fact it can be fit better for PLOS ONE journal requirement.
