# Supplementary material for: Survival outcomes of breast cancer patients with recurrence after surgery according to period and subtype
Source: PLoS One. 2023 Jul 27;18(7):e0284460. doi: 10.1371/journal.pone.0284460 (PMC10374104; doi:10.1371/journal.pone.0284460)
Supplement: Attachment — Submitted filename: Response to Reviewers.docx [file pone.0284460.s009.docx]

Yasunori Sato, Ph.D.

Academic Editor

*PLOS ONE*

01/06/2023

Dear Dr. Yanunori

On behalf of the authors, I would like to thank you and the reviewers for the valuable and constructive comments, which helped us immensely to improve our submitted manuscript, “Survival outcome analysis of patients with recurrent breast cancer after surgery according to period and subtype” (PONE-D-22-18837).

We have carefully reviewed the comments and have revised the manuscript accordingly. We have provided the responses in a point-by-point manner, and changes made in the manuscript are shown in [yellow highlight], which are also visible through the Track Changes feature.

We would be happy to make further corrections if necessary, and we look forward to hearing from you soon.

Sincerely,

Sae Byul Lee, M.D., Ph.D.

Clinical Associate Professor

Asan Medical Center, University of Ulsan College of Medicine

88, Olympic-ro 43-gil, Songpa-gu, Seoul 05505, Republic of Korea

Tel: +82-2-3010-1729, Cell Phone: +82-10-7209-4620

Email: newstar153@hanmail.net

Response to Reviewer #1

Thank you for your review of our paper and for your constructive comments. We have answered each of your points below.

Comment 1: Don’t mention the definition of period1 and period2 multiple times (lines 21,25,55, 63, 169)! Mentioning once would be enough!

Response: We appreciate the reviewer’s comment. We have now corrected this as per the reviewer's comment by mentioning the definition once in the abstract and once in the main text. (lines 25,56,64,181)

Comment 2: Your sample size is 2,407, so report it in abstract and method as your sample size as your analysis is based on that.

Response: We appreciate the reviewer’s comment. In agreement with the reviewer’s comment, we have now mentioned it in the Abstract and Method section. (lines 24,62)

*“We retrospectively analyzed 2,407 patients who had recurrent breast cancer with treated between January 2000 and December 2013 and divided them into two periods according to the year of recurrence.” (line 24)*

*“Among them, we selected 2,407 patients who had experienced recurrence before December 31, 2020.” (line 62)*

Comment 3: It seems that there is an abbreviation in the literature stands for post-recurrence survival (PRS).

Response: We appreciate the reviewer’s comment. We have now corrected this as per the reviewer's comment. (lines 57)

*“..to identify the factors associated with overall and post-recurrence survival”. (line 56)*

Comment 4: There is a lack of proper rationales in introduction part. Why this study is important?

Response: We appreciate the reviewer’s comment. The Introduction section has now been revised to include the following statement as per the reviewer’s comment.

*“**Therefore, it is necessary to analyze the impact of the development of adjuvant therapy over time for each subtype on the prognosis of relapsed patients.” (line 51)*

Comment 5: SAR should be defined from the time of recurrence to death/last follow up. OS is also defined time from diagnosis to death/last follow up time.

Response: We appreciate the reviewer’s comment. In agreement with the reviewer’s comment, we have now revised it as follows:

*“**Overall survival (OS) was defined as the time from surgery to death/last follow up. SAR was defined as the time from recurrence to death/last follow up by referring to the Korean registry cause-of-death records.” (line 70)*

Comment 6: What about eligibility criteria! Is your study restricted to age (e.g., 18-77), those who had not second recurrence or metastasis etc.

Response: We appreciate the reviewer’s comment. The age of the participants in this study was 21–88 years. Among them, 14 and 7 patients aged above 77 and 80 years, respectively, were expected to have no significant effect on the overall outcome. For more than two recurrences, data were not sufficient; therefore, we decided to analyze them in future studies.

Comment 7: If study aimed to see improvement in treatment in patients who had recurrence, the time of treatment is of importance. If so, the treatments for patients with recurrence should be considered as well! You don’t know is this the effect of treatments before recurrence or after the recurrence. This bias can affect the result. Were patients received the treatment after their recurrence? There is no information in the manuscript to support this! The treatments mentioned in table 2, are received by patients after recurrence?

Response: We appreciate the reviewer’s comment. Among the treatments listed in Table 2, if “after recurrence” is appended to the name of the treatment, it refers to the treatment applied after the recurrence, and otherwise implies the adjuvant therapy received during the first treatment. To clarify this, we have now added this information in the Methods section. Treatment after recurrence was used in Cox regression analysis.

*“All information about the patients and diseases was retrieved from the retrospectively collected database, including age; clinical manifestations; clinical and pathological staging according to the American Joint Committee on Cancer classification; pathological data; surgical methods; types of adjuvant therapy received during the first treatment; types of post-relapse adjuvant therapy, which marked the “after recurrence,” type; and follow-up period.” (line 68)*

Comment 8: In table 2, comparison results showed that in some pathologic variables, there are significant differences between two periods (e.g., T stage, Nodal status, HR etc)! It seems that you might need to match comparison groups. The question is that are they comparable?

Response: We appreciate the reviewer’s comment. If we match the variables, the results cannot be generalized. Therefore, we did not match the comparison groups. This is a limitation of our study and has been added to the Discussion section. Additionally, multivariate Cox model was used to minimize the influence of other variables.

*“**Third, there were significant differences in some pathological variables between the two periods. However, we did not match the variables to generalize the results.” (line 241)*

Comment 9: Your sample size covers recurred patients (your title), right? while you have results based on overall survival which includes interval time from diagnosis to death/last follow up!

Response: We appreciate the reviewer’s comment. Yes, our sample size covers recurred patients. Our results are based on overall survival as well as survival after recurrence.

Comment 10: Be clear on your sample size! It looks Analyses were performed based on recurred sample! If you are analyzing 17,776 patients, mention the sample of analysis in table 4 and 5.

Response: We appreciate the reviewer’s comment. We had analyzed 2,407 patients. We have now mentioned it clearly in the manuscript and have highlighted them in previous comments.

Comment 11: Why didn’t you use trend analysis to see the changes over time?

Response: We appreciate the reviewer’s comment. In this study, trend analysis was not performed because our focus was on the difference between the two periods rather than on the serial change.

Response to Reviewer #2

Thank you for your review of our paper and for your constructive comments. Our answers to your queries are as follows.

Comment 1: Authors include the patients with unknown clinical variables e.g., stage, histology, etc. Why not removing these patients? If for any clinical and biological reason, this group named as “unknown” is important and would like to assess the effect of that in analyses, still not correct analysis was applied. Authors considered chi-squared method to do the association which will have low power in this scenario, and the non-parametric Fisher exact test is the correct method to be applied.

Response: We appreciate the reviewer’s comment. The unknown groups of each variables were removed when we performed the uni/multivariate Cox regression analysis. The Methods section has now been revised to clarify this.

*“The unknown groups of each variables were removed prior to Cox analysis.” (line 98)*

Comment 2: Authors did mention that multivariate Cox model was applied, and I couldn’t follow in which step this method was applied. I already saw the findings using univariate Cox model.

Response: We appreciate the reviewer’s comment. We performed univariate Cox regression analysis to determine how the factors impact survival. Subsequently, we performed multivariate Cox regression analysis to eliminate the interaction of multiple, potentially interacting covariates. As variables, we selected the periods, age at diagnosis, adjuvant therapy after recurrence, and pathological variables that were predicted to affect survival with other covariates.

Comment 3: Please keep the HR estimate along with p value to report findings.

Response: We appreciate the reviewer’s comment. We have now revised this as per the reviewer's comment. (lines 166-171)

Comment 4: Table 3: Please add appropriate test to assess the association between type of recurrence and year of recurrence.

Response: We appreciate the reviewer’s comment. We have added chi-squared tests to assess the association.

*“There were significant differences in the type of recurrence according to the time period (p < 0.001).” (line 135)*

Comment 5: Figures 2 and 3: How about adding supplementary figures to show the KM and log-rank test using OS across all four subtypes and the same test for SAR.

Response: We appreciate the reviewer’s comment. We have added supplementary figures to show the KM and log-rank test using OS and SAR across all four subtypes.

Comment 6: Tables (e.g., 4 and 5), for example for some categorical variables e.g., age, T stage, etc., we have the p values and other statistics for each category, while a p value for that given variable (e.g., age, T stage). I couldn’t follow how the p value was computed.

Response: We appreciate the reviewer’s comment. The statistical analysis part of the Methods section has now been revised as per the reviewer’s comment.

*“A multivariate Cox regression analysis with a backward elimination method was used to estimate the hazard ratios and p values and to identify independent prognostic factors.” (line 96)*

Comment 7: In addition, authors only considered clinical data, while adding molecular data along with clinical data to assess the association with OS or SAR can make the paper much more interesting along with this fact it can be fit better for PLOS ONE journal requirement.

Response: We appreciate the reviewer’s comment. As of now, collecting molecular data is very challenging. As a follow-up study, we will consider the analysis of molecular data in the future.

Response to Reviewer #3

Thank you for your review of our paper and for your constructive comments. We have answered each of your points below.

Comment 1: Using anti-hormonal therapy instead of hormonal therapy is not common. Even though It is not incorrect, it is suggested to change it.

Response: We appreciate the reviewer’s comment. We have revised the terminology as per the reviewer’s comment.

Comment 2: Table 3: According to data in table 3, no patient had a locoregional and systematic recurrence. While the co-incidence of outcomes is possible.

Response: We appreciate the reviewer’s comment. There were 615 patients who experienced locoregional and systemic recurrence simultaneously. We have included this information in the “systemic recurrence” category.

Comment 3: Line 101: You have presented that the follow-up duration from the time of relapse ranged between 0–223.4 months. Since the data is pertaining to 2000-2013 and they have been recruited into the database from 2017 to 2021, one expects that their follow-up time is not zero. Would you please explain more about this data?

Response: We appreciate the reviewer’s comment. There were some patients who were lost to follow up. Their follow-up duration from the time of relapse was counted as zero.

Comment 4: Please define the statistical tests which were used in the analysis of data presented in lines 131-135.

Response: We appreciate the reviewer’s comment. The median survival duration is the duration at which the probability of survival equals 50%. Survival curves were generated using the Kaplan–Meier method, and the significance was verified using the log-rank test.

Comment 5: Line 134-135: In this sentence, “… the median 5-year OS rate 135 from the period I (97.5 months) to period II (114.4 months)”, you have not presented any survival rate, and the sentence needs an edition. Those values are the median survival durations.

Response: We appreciate the reviewer’s comment. We have now corrected it as per the reviewer's comment.

*“The median survival duration after recurrence significantly increased from* *38.0 months in period I to 49.7 months in period II (p < 0.001). In contrast, the increase in the median survival duration from period I (97.5 months) to period II (114.4 months) was not statistically significant (p = 0.092; Fig 1).” (line 144-147)*

Comment 6: Tables 4 and 5 are huge boxes of data and are hard to use. The p-values can be deleted, and the significant HR (0.95 CI)s can be bolded.

Response: We appreciate the reviewer’s comment. We have now revised Tables 4 and 5 as per the reviewer’s comment.

Comment 7: Line 191: regarding this sentence and the following explanation, “In multivariate analyses, age at diagnosis was independently associated with…” we should notice that independent association of variables is studied in univariate analysis. In multivariate analysis, the effect of each variable is dependent on the effect of other included variables in the regression model.

Response: We appreciate the reviewer’s comment. We have reviewed this part again and revised it accordingly.

*“In multivariate analyses, age at diagnosis was associated with SAR and OS only in the HR-positive/HER2-negative subtype (Tables 4, 5).” (line 203)*

Comment 8: Line 208: “chemotherapy after recurrence was significantly associated with survival outcomes…”. It is not clear whether it was associated with better or worse survival?.

Response: We appreciate the reviewer’s comment. We have now corrected it as per the reviewer's comment.

*“In the analysis of treatment after relapse, chemotherapy after recurrence was significantly associated with poor survival outcomes in all subtypes (Tables 4, 5).” (line 219)*

Once again, thank you for reviewing our manuscript in detail and providing helpful comments allowing us to improve our manuscript. We hope that our responses and the corresponding revisions are satisfactory.
